# Improving prevention, monitoring and management of diabetes among ethnic minorities: contextualizing the six G’s approach

**DOI:** 10.1186/s13104-017-3104-9

**Published:** 2017-12-28

**Authors:** Anil Gumber, Leher Gumber

**Affiliations:** 1 Faculty of Health and Wellbeing, Sheffield Hallam University, Sheffield, UK; 2 Brighton and Sussex Medical School, Brighton, UK

**Keywords:** Diabetes management, Ethnic minorities, Six G’s framework, Barriers to access, Lifestyle behavioural change

## Abstract

**Objective:**

People from Black, Asian and Minority Ethnic (BAME) groups are known to have an increased risk of developing diabetes and face greater barriers to accessing healthcare resources compared to their ‘white British’ counterparts. The extent of these barriers varies by demographics and different socioeconomic circumstances that people find themselves in. The purpose of this paper is to present and discuss a new framework to understand, disentangle and tackle these barriers so that improvements in the effectiveness of diabetes interventions for BAME communities can be achieved.

**Results:**

The main mediators of lifestyle behavioural change are gender, generation, geography, genes, God/religion, and gaps in knowledge and economic resources. Dietary and cultural practices of these individuals significantly vary according to gender, generation, geographical origin and religion. Recognition of these factors is essential in increasing knowledge of healthy eating, engagement in physical activity and utilisation of healthcare services. Use of the six G’s framework alongside a community centred approach is crucial in developing and implementing culturally sensitive interventions for diabetes prevention and management in BAME communities. This could improve their health outcomes and overall wellbeing.

## Introduction

Health inequalities have existed within the UK for the past several decades with ethnic minority groups continuing to report a higher incidence of morbidity and mortality than the general population for a range of chronic diseases [1, 2]. Black, Asian and Minority Ethnic (BAME) groups have a higher risk of diabetes [3] and experience greater barriers in the healthcare-seeking process. The main barriers that have been identified include generational, geographical and gender differences within specific ethnic minority groups. Also, the presence of a profound language barrier, lack of culturally appropriate information and poorer knowledge are deemed to be other contributing factors [4]. The aims of this paper are: to discuss these underlying barriers specifically for the management of diabetes within BAME groups; and to provide a new framework to ease these barriers and improve the effectiveness of health interventions among BAME. There will be a particular focus on South Asians as they form the largest group amongst BAME within the UK [5].

## Main text

### The six G’s framework

**Fig. 1:**
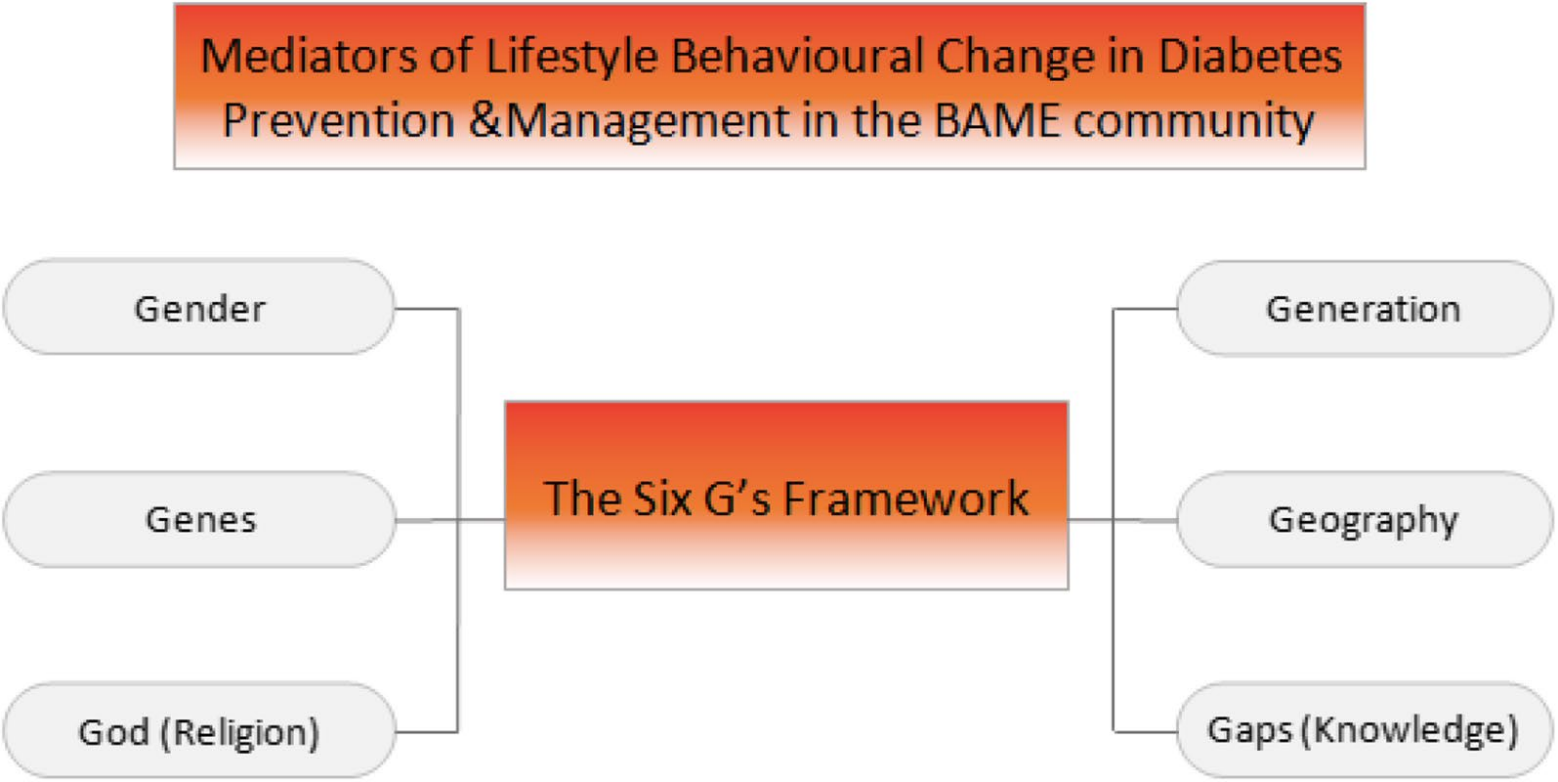
Diversity, complexity and inclusion of BAME people for health behavioural change

The proposed six G’s framework (as shown in Fig. 1) is a tool devised to be used by the healthcare professionals to enhance their client relationship with BAME communities and improve the effectiveness of health delivery services. Furthermore, it will enable new interventions to be developed that are consistent with their religious and cultural practices. This will allow the provision of additional support to individuals with long-term health conditions including diabetes to improve their overall wellbeing.

#### Gender

Self-management of diabetes poses a challenge as it places significant physical, emotional and financial burden on the affected individuals. Gender differences in knowledge and attitude will shape the methods used to control the progression of diabetes. Within the family, gender dynamics play an important role and influence household decisions. Traditionally, women from BAME communities hold the primary responsibility for child upbringing and managing household chores. However, decisions about dietary practices arise as a result of negotiations between all members of the household including men, younger and older generations. Therefore, changes to the traditional cooking and serving of food can be difficult [6].

Exercise is another lifestyle factor that is heavily influenced by gender inequalities. In general, BAME groups have been reported to engage in lower levels of physical activity compared to their white group [7]. Cultural and religious constraints can reduce these levels even further particularly when it involves women. For example, the Muslim women may not be able to participate in certain activities due to a lack of female-only classes with female instructors [8–10]. This can hinder management of diabetes and other long-term conditions [6].

Ineffective communication skills are another concern in women from BAME groups. The presence of a language barrier particularly in first generation migrants means that children and other family members are used as informal interpreters in clinical appointments. This can lead to feelings of guilt in patients and thus reluctance in seeking advice from healthcare professionals [9, 11–13]. Provision of formal interpreters may relieve the pressure on other family members and encourage attendance to clinical appointments. Gender inequalities within these communities provoke difficulties in engagement and management of health conditions. The use of family centred interventions may be more suitable in enabling women and men to adopt more positive health behaviours.

#### Generation

A generational difference within BAME groups creates variation in attitudes and beliefs of individuals. A childhood in the UK exposes second-generation migrants to different experiences through school and media compared to that of their parents. This will affect their engagement in positive health behaviours. Despite this, the morbidity and disease prevalence rates by generation are much narrower in BAME groups when compared to their white British counterparts.

In most communities, traditional food and its preparation are passed down through generations. A comprehensive review of qualitative studies on BAME people with diabetes uncovered important differences in dietary practices of first and second generation South Asians [6]. For example, the older generation prefers to eat traditional food every day and consider deviating away from this diet as an insult to their culture. Conversely, the younger generation enjoys having takeaways and eating English foods. Similar differences have been found in the levels of physical activity [14]. In a study based on the South Asian community, first generation migrants were found to be more conservative compared to the second generation in the type and level of exercise carried out [15]. The younger generation was also reported to take their children for swimming. A study carried out in the Netherlands identified variations in cardiovascular disease risk factors solely based on generational differences [16].

Poor linguistic competence is another major barrier, particularly in first generation migrants. Bangladeshis are the most affected compared to other ethnic minorities due to lower levels of education [9]. However, when registered with a GP from a similar background, these barriers are significantly reduced [17].

#### Genes

Genetic factors play an important role in susceptibility to long-term conditions. Various genes have been identified which predispose ethnic minority groups to diabetes. Genetic variants of TCF7L2 have been associated with an increased risk of type 2 diabetes in UK South Asians [18]. Similarly, the presence of HLA-DQA1 and HLA-DQB1 alleles in black Africans is noted to increase susceptibility to insulin-dependent diabetes [19]. Family history has also been identified as a risk factor for the development of diabetes and cardiovascular disease. South Asians with diabetes were found to be 52% more likely to develop type 2 diabetes if a family history was present compared to 41% in white Europeans [20].

#### Geography

It is widely recognised that prevalence of cardiovascular disease and diabetes in minority ethnic groups is significantly greater compared to UK’s general population [21, 22]. One of the greatest challenges in devising appropriate screening and intervention programmes is the vast differences in culture within this community. Ancestral roots in these groups vary from Kenya and Uganda to South Asian countries (India, Pakistan, Bangladesh and Sri Lanka). Country of origin and migration history has a major effect in shaping health-related behaviour and beliefs. It influences their organisation of family, language and cultural practices in the UK [23].

#### God (religion)

The South Asian community living in the UK is heterogeneous with diverse religions [24]. Religious beliefs and practices play an important role in the diagnosis and daily management of a long-term condition. The conventional approach to preventing disease through lifestyle modification is difficult to apply in ethnic minorities. Thus the implication of religion on behaviour is an important factor to consider when engaging with these communities [25]. In several studies when asked about the cause of diabetes, participants have reported that it is due to God’s will. They believe that the disease has occurred as a result of their sins and thus accept it with resignation. This can form a major barrier preventing individuals from adhering to treatment recommendations [6, 25]. Similar beliefs were also reported by Muslims for cancer screening and treatment [26].

Furthermore, religious practices can influence dietary attitudes. Many places of worship including Mosques, *Gurudwaras* (Sikh temples) and Hindu temples serve free meals and provide opportunities to socialise and eat together. The preparation of food in places of worship follows the traditional diet which is rich in fat, dairy and sometimes meat [27]. This can form an obstacle when adopting a low fat and carbohydrate diet and even lead to alienation from the community.

Religious rituals and fasting are very common in the South Asian community. This may be on a regular basis or as part of a special observance such as during Ramadan among Muslims. Alterations in diet, timings and levels of activity during these periods can have a significant impact on diabetes and health management [21].

#### Gaps in knowledge and economic resources

Knowledge of positive health behaviours relating to BMI, smoking, physical activity and nutrition in ethnic minority communities is still a major concern. Many South Asians underestimate the importance of eating fruits and vegetables. In-depth interviews with South Asian women with diabetes revealed that bananas, apples, and potatoes were seen as raising blood glucose levels and hence were not consumed. This perception may cause challenges in adhering to the recommended 5-a-day guidelines [17]. Using revised WHO cut-offs for Asian populations for obesity [28], it was found that 80% of South Asians with diabetes were obese when compared to only 55% of white Europeans [20]. Furthermore, the majority of BAME people lives in an economically deprived area and engaged in low-paid occupations [29]. The Department of Health report highlighted a wide and persistent economic gap over the last three decades in terms of lower employment rates in BAME people compared to white British [30]. A low income and socio-economic status in ethnic minorities has a direct impact on affordability and accessibility to relevant healthcare services and is reflected in a considerably lower uptake of health screening services [31]. It was observed that 49% of South Asians with diabetes compared to 7% of white Europeans were living in areas of high economic deprivation [20]. Thus, low levels of knowledge (e.g. education) and economic resources (e.g. income and house ownership) augment further barriers to effective engagement with these communities.

### Implications for future practice

The increase in the incidence of chronic diseases particularly diabetes in ethnic minorities creates a serious challenge for healthcare providers. It is imperative that healthcare professionals, policy makers, and commissioners recognise the diversity of beliefs and cultural practices within these groups and ensure all interventions are tailored according to their specific ethnicity, age, religious beliefs and cultural needs. More importantly, adopting a community-centred approach is essential in improving their health and wellbeing. In a large clinical trial for South Asian people with diabetes, a successful community diabetes-specialist nurse/link worker intervention on dietary and lifestyle behavioural changes was specifically tailored to diverse participants in terms of gender, generation, religion, language and geographical origin [32].

Local projects working in partnership with places of worship should be initiated to increase awareness of the risks associated with their lifestyle practices and promote behavioural change. The benefits of this were demonstrated in a project by Apnee Sehat who worked alongside a Gurudwara to encourage lifestyle modifications. Participants reported that they made changes both at an individual and household level [33]. Furthermore, any dietary advice that is given should be culturally sensitive. For example, the older generation may require guidance on how to make amendments to traditional recipes which are still deemed to be acceptable by other household members. In contrast, the younger generation may benefit from more westernised interventions. Similarly, encouraging regular exercise and referral to gender-specific exercise classes/facilities by GPs particularly for BAME women may motivate them to engage in lifestyle modifications. There are notable examples of BAME community trained volunteers/health champions involvement in community engagement to promote physical activity through enjoyable exercise and health education which could be replicated elsewhere. In the Tandrusti Project of Dudley, after attending exercise and health educational classes, the BAME participants from diverse backgrounds (gender, generation, religion, income, and education) with long-term conditions including diabetes, had reported significant changes in blood pressure, obesity and other metabolism outcomes and recorded improvements in their health, fitness, self-esteem, quality of life and wellbeing as well as boosted their confidence in accessing health services independently [34].

### Conclusion

It is paramount that all aspects of the six G’s framework be implemented to reduce barriers and optimise outcomes and effectiveness of health interventions for ethnic minorities. The application of this framework has shown encouraging results for enhanced community engagement and active participation by BAME people in adapting healthy lifestyle changes and improvement in quality of life and wellbeing. This framework/tool could enhance cultural competence among healthcare professionals for better management of long-term health condition affecting ethnic minorities such as cardiovascular disease, cancer, stroke, and diabetes. It could also be integrated into the MRC guidance for development of complex interventions to improve health [35] through health behaviour change programmes specifically targeting BAME communities.

## Limitations

The framework/tool is presented with healthcare service provider perspective which needs further testing and applicability in the wider settings.
